# Can Dynamic Computer‐Guided Surgery Be Useful for Removing an Upper Jaw Odontoma?

**DOI:** 10.1002/ccr3.70709

**Published:** 2025-09-22

**Authors:** Martina Mezio, Davide Brilli, Alessandra Putrino, Matteo Giansanti, Michele Cassetta

**Affiliations:** ^1^ Department of Oral and Maxillofacial Sciences Sapienza University of Rome Rome Italy

**Keywords:** canine impaction, dynamic computer‐guided surgery, miniscrew, odontoma, TADs

## Abstract

Dynamic navigation systems can improve precision and accuracy in oral surgical procedures. Odontomas are benign lesions commonly encountered in clinical practice. When asymptomatic, they are often discovered in cases of persistence of deciduous elements, and the permanent teeth fail to erupt spontaneously. The surgical removal approach is the gold‐standard treatment, but is not without risks. This case report presents a 14‐year‐old boy with an impacted upper canine in the presence of an odontoma near the roots of the permanent central and lateral incisors. The removal of the odontoma and the recovery of the canine were performed using a dynamic navigation system. Integrating the navigation system with cone beam computed tomography (CBCT) allowed for precise planning and real‐time guided surgery, ensuring patient safety, comfort, and predictability. This approach highlights the potential of dynamic navigation systems not only for implant rehabilitation but also for advancing oral surgery procedures, paving the way for future developments in dentistry that can better leverage the clinical skills of operators.

**Summary**

- The integration of computer‐guided dynamic surgery with conventional surgical techniques enables real‐time surgical guidance and accurate odontomas removal, enhancing procedural safety and minimizing intraoperative risks.
- This approach could significantly improve patient comfort, preserve adjacent structures, and potentially reduce recurrence rates, ensuring predictable results.

## 1 Introduction

Odontomas are among the most common benign odontogenic tumors, often discovered during routine dental imaging due to their asymptomatic nature. They are typically classified as either compound or complex odontomas: compound odontomas resemble normal tooth structures, while complex odontomas appear as disorganized masses of dental tissues, including enamel, dentin, and cementum [1, 2]. Although the exact etiology of odontomas is unclear, factors such as dental trauma, infection, and genetic predisposition are thought to play a role in their development. These tumors primarily affect children and young adults, often disrupting normal tooth eruption, especially when located near permanent or deciduous teeth [3, 4, 5]. Surgical removal is generally the preferred treatment, as it helps to prevent eruption complications and allows for further orthodontic intervention if needed [6, 7, 8, 9, 10].

Recent advancements in dentistry focused on dynamic navigation systems (DNS) as a groundbreaking technology aimed at enhancing the accuracy and efficiency of implant procedures [11, 12]. DNS relies on precise motion tracking and 3D visualization to guide the virtual planning and execution of surgeries. By using a computer, specialized navigation software, and synchronization with external tracking tools, the system provides real‐time guidance to the operator [13]. This implies the application of a workflow that begins with the acquisition of preoperative data, followed by imaging data processing and 3D reconstruction. Real‐time guided surgery then follows, after surgical instrument calibration and spatial recognition [14]. Compared to traditional freehand techniques, dynamic navigation systems offer improved efficacy and accuracy in dental implantology, also helping clinicians improve their surgical skills [15]. Over time, this technology has expanded beyond implantology [16], with applications in various oral and maxillofacial surgeries, including dental extractions, tumor resections, fracture repairs, temporomandibular joint ankylosis treatments, and orthognathic surgeries. It has also been used in intraosseous anesthesia and endodontics [17, 18, 19, 20, 21]. Dynamic guided surgery systems help to reduce procedural times, improve patient outcomes, and increase satisfaction while minimizing complications during various dental procedures [11, 12, 13]. This technology not only enhances surgical accuracy but also contributes to a more predictable workflow, reducing operator stress and improving overall clinical efficiency.

This case report was conducted, following the CARE guidelines, to present the use of a dynamic navigation system in the surgical removal of an odontoma and the recovery of an unerupted canine in the maxillary arch of a young patient.

## 2 Case History and Examination

**FIGURE 1:**
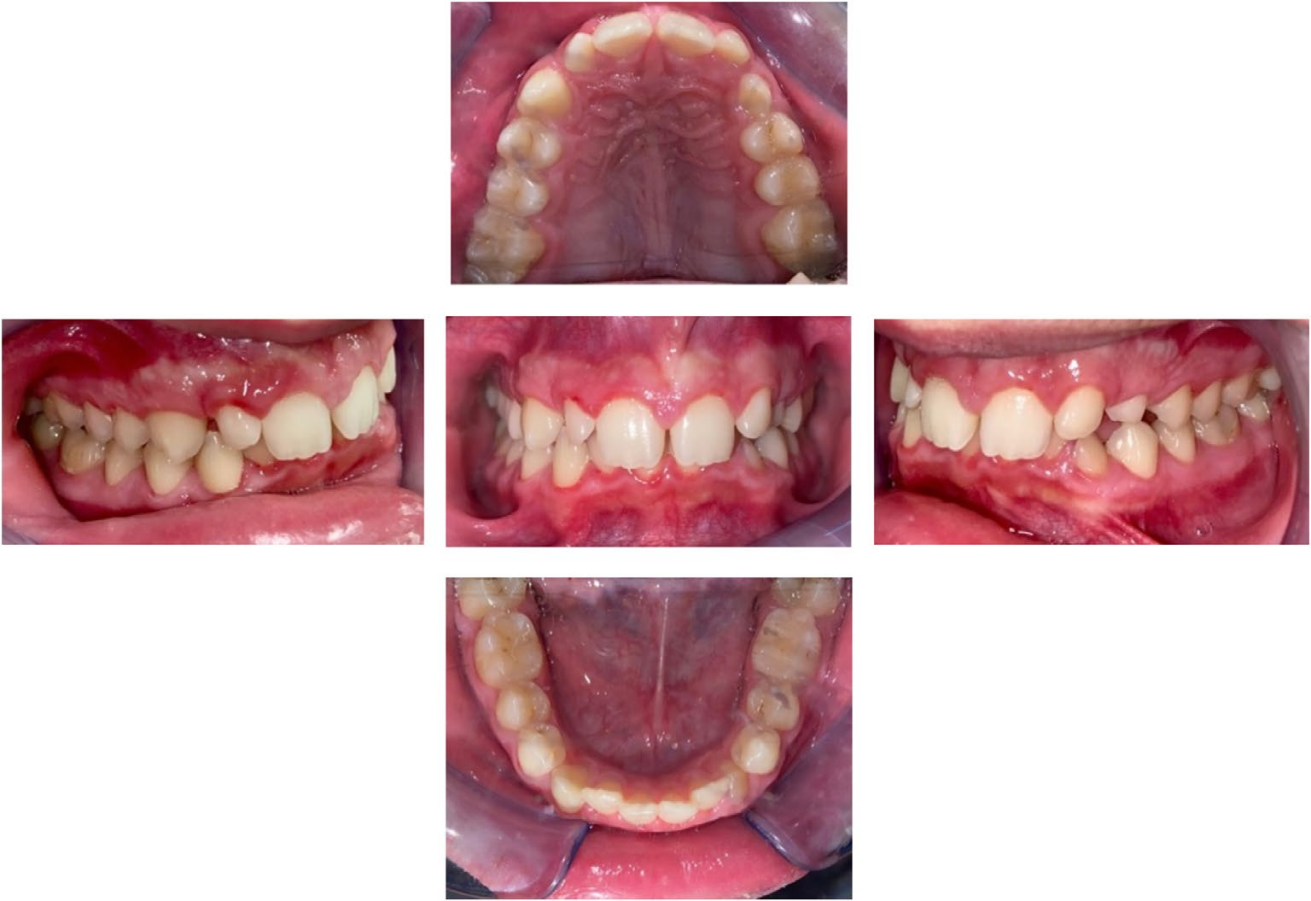
Pre‐treatment intraoral photographs.

**FIGURE 2:**
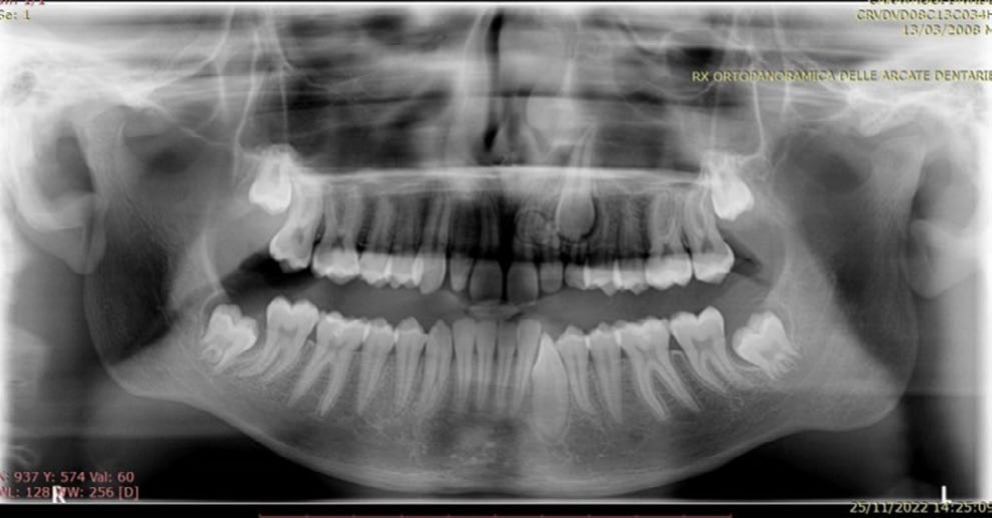
Pre‐treatment orthopantomography.

**FIGURE 3:**
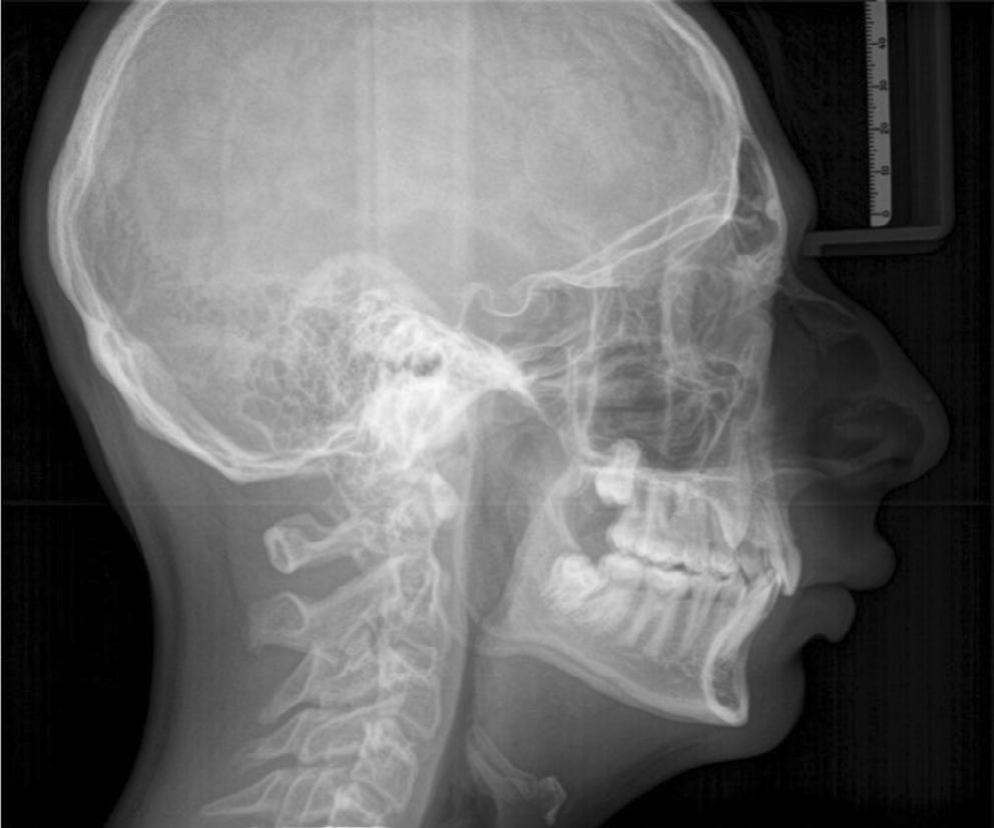
Pre‐treatment lateral cephalogram.

A 14‐year‐old male patient presented for evaluation due to the persistence of the upper left deciduous canine and the management and concerns about his dental and skeletal malocclusion. Clinical examination revealed a late mixed dentition with the persistence of a deciduous canine on the left side, a class I molar relationship, a deep bite, midline deviation, and mild dental crowding in the lower arch (Figure 1). Radiographic records showed an impacted permanent left canine and a corresponding osteodense lesion adjacent to the root surfaces of the central and lateral incisors as well as the crown of the impacted canine (Figure 2). The vitality of the central and lateral incisors was preserved. The lateral cephalogram showed class II skeletal malocclusion, decreased divergence with a pattern of horizontal growth, along with altered dental parameters related to incisal inclination (Figure 3, Table 1).

**TABLE 1:** Cephalometric values (Oris Ceph, Elit Computer 8.3.1, Vimodrone, Milan, Italy).

Outcome	Mean	Value
SNA	82° ± 2°	80.79°
SNB	80° ± 2°	73.81°
ANB	2° ± 2°	6.98°
Wits Appraisal	0 ± 2 mm	6.34 mm
BasSN	130° ± 4°	129.89°
SN‐GoMe	32° ± 3°	28.62°
Gonial angle	126° ± 4°	110.15°
Upper gonial angle	52° ± 3°	42.73°
Lower gonial angle	72° ± 3°	67.42°
FMA	26° ± 3°	14.58°
Anterior facial height	102 mm	124.27 mm
Posterior facial height	66 mm	87.61 mm
Facial height ratio	60%	70.50%
Ramus height	42 mm	52.14 mm
Mandibular body length	—	76.07
Bjork sum	396° ± 6°	388.63°
Interincisal angle	131° ± 5°	136.63°
U1 to maxillary plane	109° ± 4°	104.70°
IMPA	90° ± 5°	102.92°
Nasolabial angle	95° ± 10°	91.98°
Upper lip to E‐plane	−2/−4 mm	4.03 mm
Lower lip to E‐plane	0/−2 mm	2.17 mm

**FIGURE 4:**
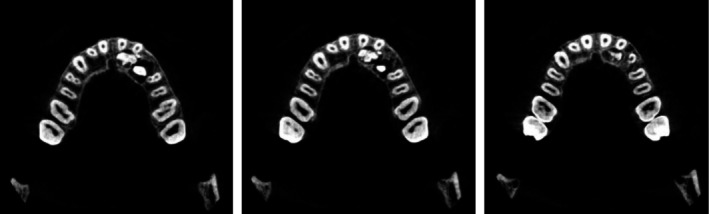
CBCT sections showing the presence of the odontoma and its proximity to permanent teeth.

The presence of the unclear lesion necessitated a CBCT scan. Given the anatomical complexity of the case, a three‐dimensional assessment was crucial to define the exact location and extent of the lesion, allowing for a precise surgical strategy. The tomographic examination confirmed the diagnosis of odontoma located in the palatal position, with anatomical proximity to the roots of the incisors and the crown of the impacted canine (Figure 4).

## 3 Treatment Objectives

The main treatment objective was to execute a full‐thickness flap in order to remove the odontoma, perform traction of the impacted canine, and sequentially activate traction of the impacted canine using a skeletal anchorage device. This approach aimed to minimize the risk of damage to adjacent structures while ensuring a controlled and predictable repositioning of the impacted tooth.

The surgical treatment plan was divided into several phases. The priority was the surgical removal of the odontoma. This was followed by a phase of surgical‐orthodontic anchoring of the impacted canine using a bone‐borne appliance designed with two palatal mini‐screws, aimed at achieving both aesthetic and functional recovery. The next phase involved active orthodontic traction of the canine with the personalized device, while simultaneously addressing the underlying dental and skeletal malocclusion through fixed multibracket therapy.

The use of a guided dynamic surgery system, Navident (ClaroNav, Toronto, Ontario, Canada), for the surgical removal of the odontoma provided a valuable opportunity to better plan and execute the procedure. Given the lesion's proximity to the impacted canine and the root tips of the central and lateral incisors, structures at significant risk of damage, this system allowed for enhanced precision. The choice to use dynamic navigation systems was driven by the opportunity to view DICOM images in real‐time, during the ostectomy and lesion removal, to reduce iatrogenic damage such as injury to adjacent teeth. Additionally, the drill (tip) or the “tracer” of the dynamic system acted like a “probe” allowing the surgeon to confirm complete enucleation of the lesion; therefore, reducing the risk of recurrence and eliminating the need for post‐radiographic verification. The subsequent treatment plan, involving a skeletal anchorage traction device and fixed orthodontic therapy rather than clear aligners, was based on the clinical expertise and experience of the operators, ensuring optimal management and predictable results.

## 4 Treatment Alternatives

Several treatment alternatives were evaluated for the resolution of this case report, such as:

- Maintaining the odontoma and the impacted canine, with regular long‐term monitoring. This approach would require periodic clinical and radiographic follow‐ups to assess potential odontoma growth or changes in the canine's position. If complications arise over time, surgical intervention may still be necessary.
- Extraction of the impacted canine while preserving the deciduous canine. This option avoids the complexity of surgical exposure but carries the risk of deciduous canine resorption, potentially necessitating future prosthetic or implant rehabilitation.
- Extraction of the deciduous canine and autotransplantation of the impacted canine. A more complex approach dependent on anatomical and biological factors, with uncertain outcomes regarding the viability and stability of the transplanted tooth.
- Removal of the odontoma and canine traction with traditional dental anchorage using conventional dental anchorage (such as brackets and bands on adjacent teeth) to guide the impacted canine into position was considered. However, this method posed several challenges, including undesired tooth movements, increased biomechanical complexity, and prolonged treatment duration. Due to these limitations, skeletal anchorage with palatal miniscrews was preferred to ensure greater biomechanical control and a more predictable outcome.
- Freehand removal of the odontoma and canine traction without computer‐assisted surgery. The odontoma could have been removed using a freehand surgical approach without dynamic navigation. While this method relies on the surgeon's expertise and preoperative imaging, it lacks real‐time guidance, increasing the risk of iatrogenic damage to adjacent structures.

After evaluating these options, the patient's parents opted for a computer‐assisted approach, integrating dynamic navigation for odontoma removal, skeletal anchorage with miniscrews, and subsequent orthodontic treatment for proper alignment. This decision was influenced by the need for a highly precise surgical approach, reducing iatrogenic risks and optimizing post‐operative recovery.

## 5 Treatment Progress

Written informed consent was obtained from the parents to proceed with the surgical removal of the odontoma, insertion of orthodontic mini‐screws, surgical intervention for canine disimpaction, and further, for the orthodontic treatment. The treatment was conducted following the ethical principles of the Declaration of Helsinki.

### 5.1 Planning Phase

**FIGURE 5:**
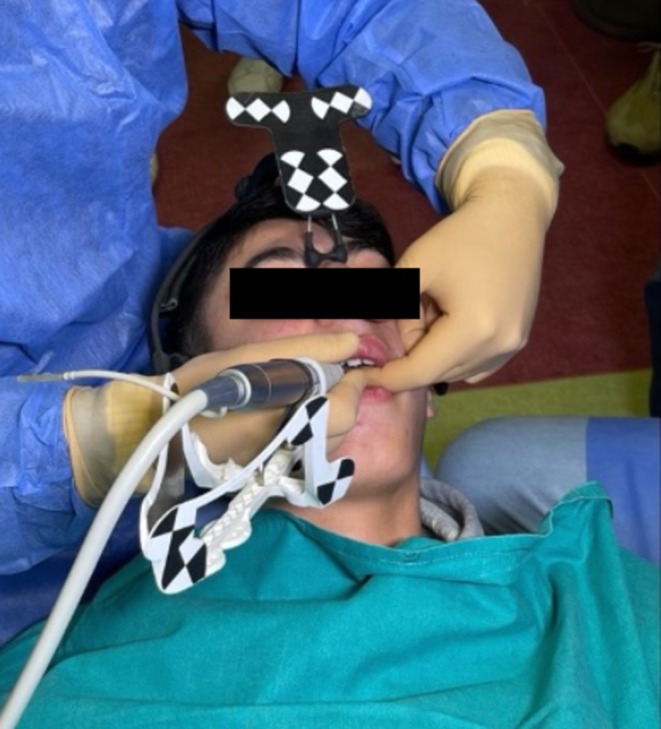
The use of Navident system on the patient.

**FIGURE 6:**
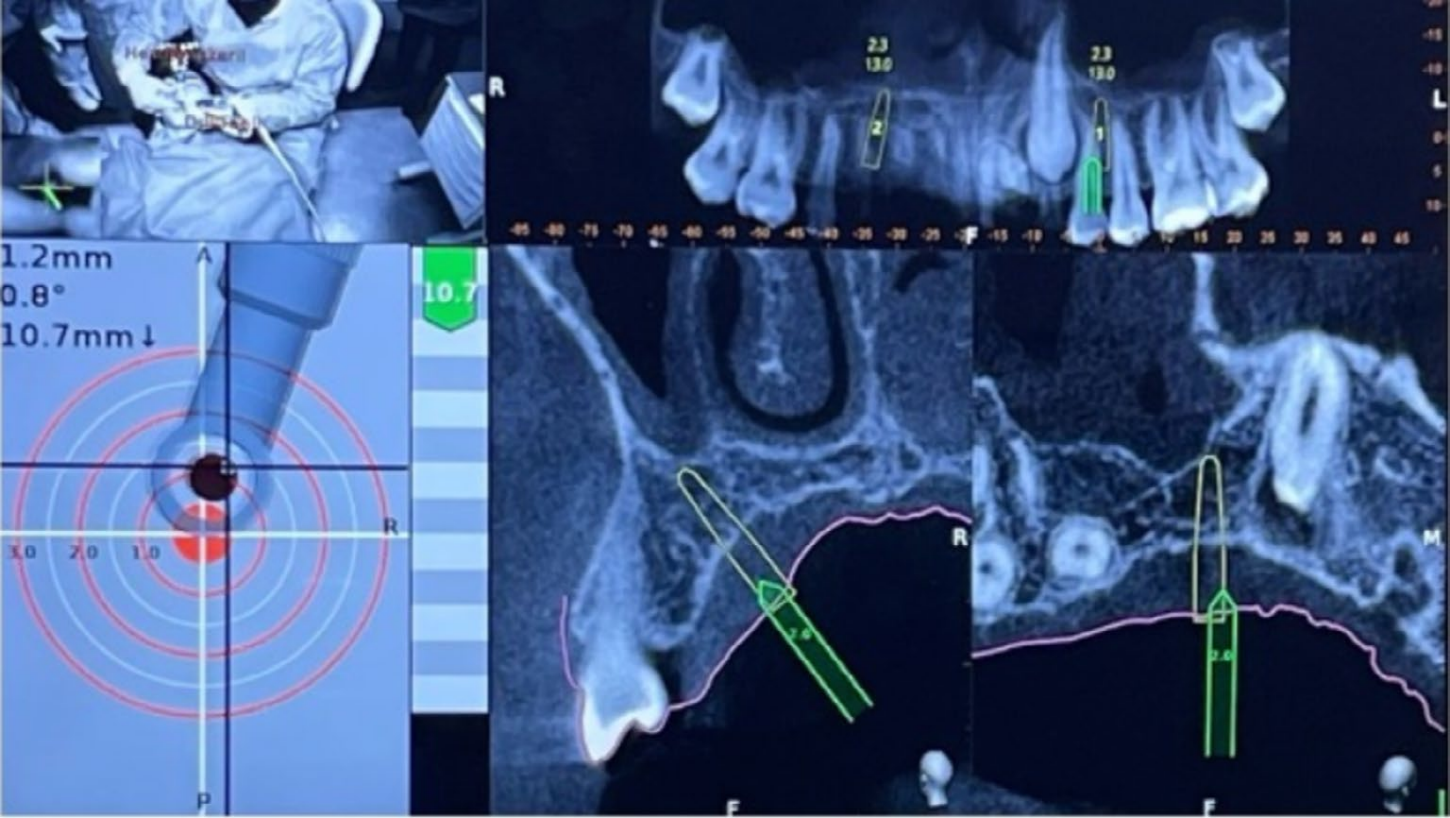
Navigation view during the insertion of a paramedian orthodontic miniscrew.

The availability of the CBCT, requested for in‐depth diagnostic analysis, enabled the pre‐operative planning for the removal of the odontoma and the development of a personalized disimpaction device. Specially, to obtain skeletal anchorage for the orthodontic treatment, two miniscrews were inserted under local anesthesia in the paramedian region at the level of the third palatine rugae. This procedure was performed by a single clinician (M.C.) an expert in both free‐hand and s‐CAS (static‐computer aided surgery), employing bicortical anchorage. The safe and minimally invasive flapless insertion has been performed without the traditional pre‐drilling step but using directly the Navident computer‐guided dynamic navigation system (ClaroNav Technology Inc., Toronto, Canada). The miniscrew insertion was conducted using a NSK iSD900 cordless battery screwdriver (Nakanishi INC., Tochigi, Japan) (Figures 5 and 6).

The intra‐oral scanning (Medit i700, Medit Corp., Seoul, South Korea) of the upper dental arch and the CBCT examination (Scanora 3Dx cone‐beam device, Soredex, Tuusul, Finland) of the upper jaw allowed obtaining respectively STL (Standard Triangulation Language) and DICOM (Digital Imaging and COmmunications in Medicine) files.

They were then superimposed to determine the design of the personalized orthodontic device and the insertion sites.

The titanium self‐drilling miniscrews (Benefit system; PSM Medical Solutions, Tuttlingen, Germany) used in the procedure had a diameter of 2.3 mm and a length of 13 mm. Both the diameter and length of the miniscrews were pre‐determined in this planning phase based on bone quantity to ensure bicortical anchorage.

### 5.2 Surgical Phase

**FIGURE 7:**
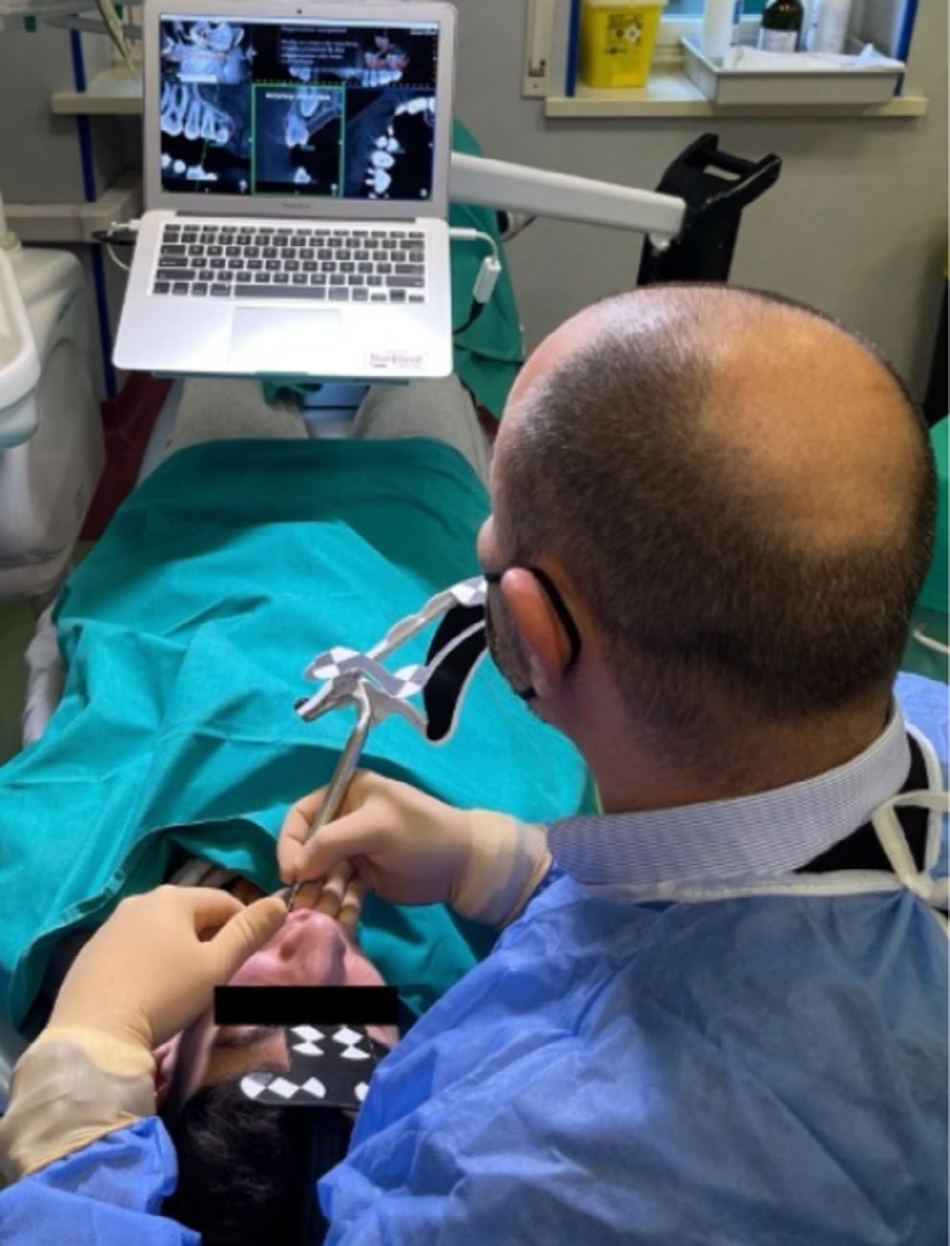
Trace registration and accuracy assessment using the tracer instrument.

The surgical phase began by placing a reference pattern tag called “Head tracker” temporarily fixed to the head of the patient. The subject was then instructed to rinse with 0.2% chlorhexidine mouthwash for 1 min preoperatively. Plexus anesthesia was performed using mepivacaine with adrenaline 1:100.000 (Molteni Dental SRL, Milano, Italy). Next, the calibration phase was performed by selecting three reference points from the three‐dimensional reconstructed DICOM images. Using a tracer instrument, these points were located in the dental arches. This procedure allowed for registration of the maxilla's relative position. (Figure 7) The Navigation Workflow phases are described in Table 2.

**TABLE 2:** Explanation of clinical steps in the use of dynamic navigation systems.

Phase	Explanation
Scan Stage	Execution of the oral scanning and CBCT examination
2 Plan Stage	The DICOM files obtained from the CBCT are matched with the STL files obtained from the intraoral scan. In this phase the insertion site, length and diameter of the miniscrew is choose
3 Register Stage	The head tracker is fixed on the head of the subject. Furthermore, three teeth are traced with the tip of the “tracer” instrument in order to generate a 3D volumetric mesh by the software. In this way the position of the patient's jaw is matched with the virtual mesh The final step involves the calibration of the handpiece and, finally, the calibration of the miniscrew installed on it The surgical phase should start only when the software confirms an acceptable accuracy (≤ 0.5 mm)
4 Treatment Stage	The software provides a “bull‐eye” visual indicator showing the deviation between the plan and the current position of the screw, allowing the operator to adjust the position of the insert, enabling an instantaneous and real‐time guidance throughout the intervention

The insertion of the two miniscrews required for orthodontic surgical traction was the first step in the surgical procedure and was performed using the flapless technique, allowing a minimally invasive approach and reduced morbidity.

This was followed by the preparation of a full thickness intrasulcular flap, extending from the second left premolar to the upper central incisor, to expose the area of the odontoma.

**FIGURE 8:**
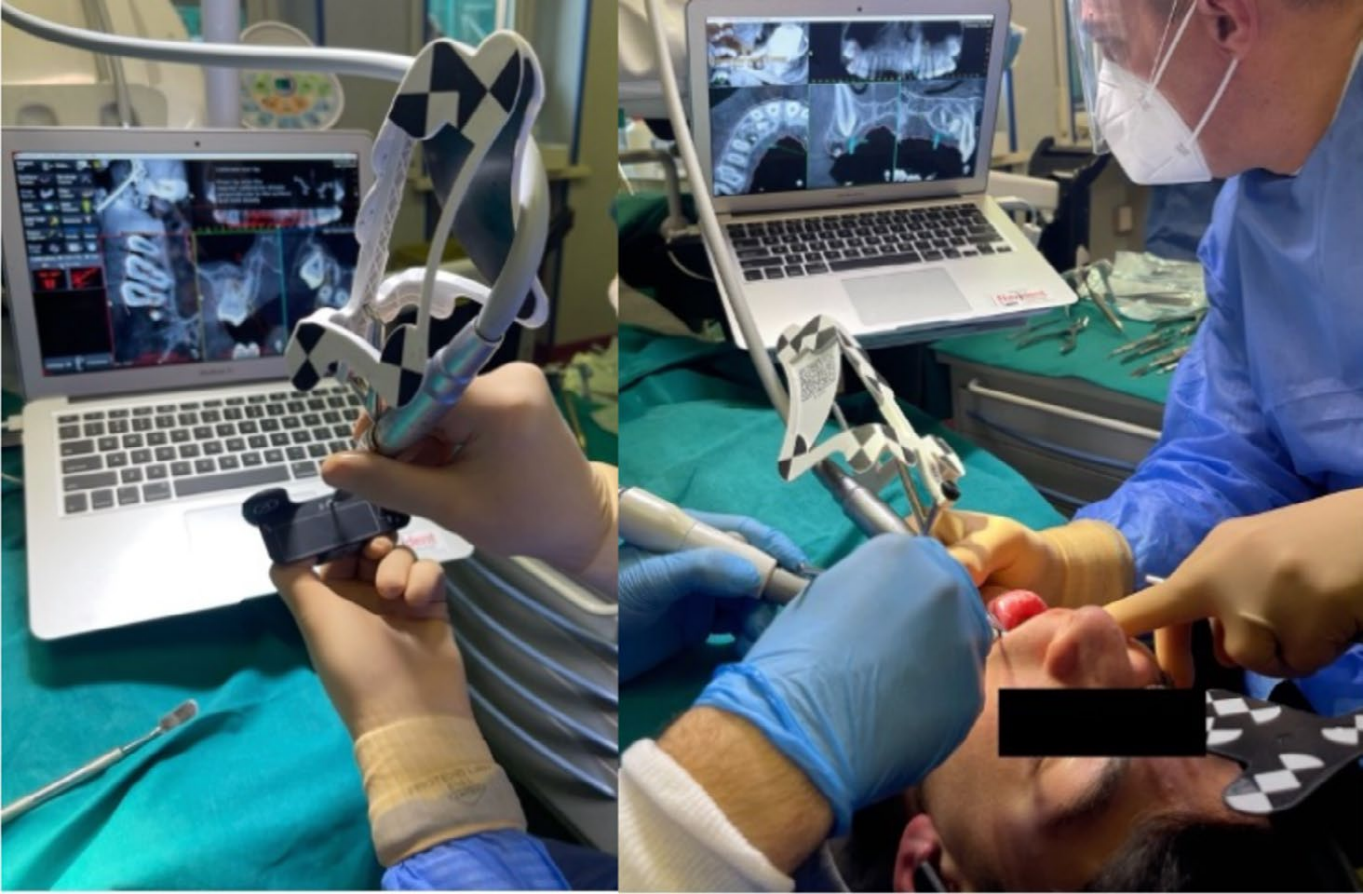
Calibration of the rose‐head bur. Use of the navigation system to guide the execution of ostectomy. Blue arrow indicates in real‐time the exact position of the instrument tip.

To accurately identify the position of the odontoma, the dynamic navigation system was initially used with a rose‐head bur, employing it as an “inspection probe” after calibration (Figure 8).

**FIGURE 9:**
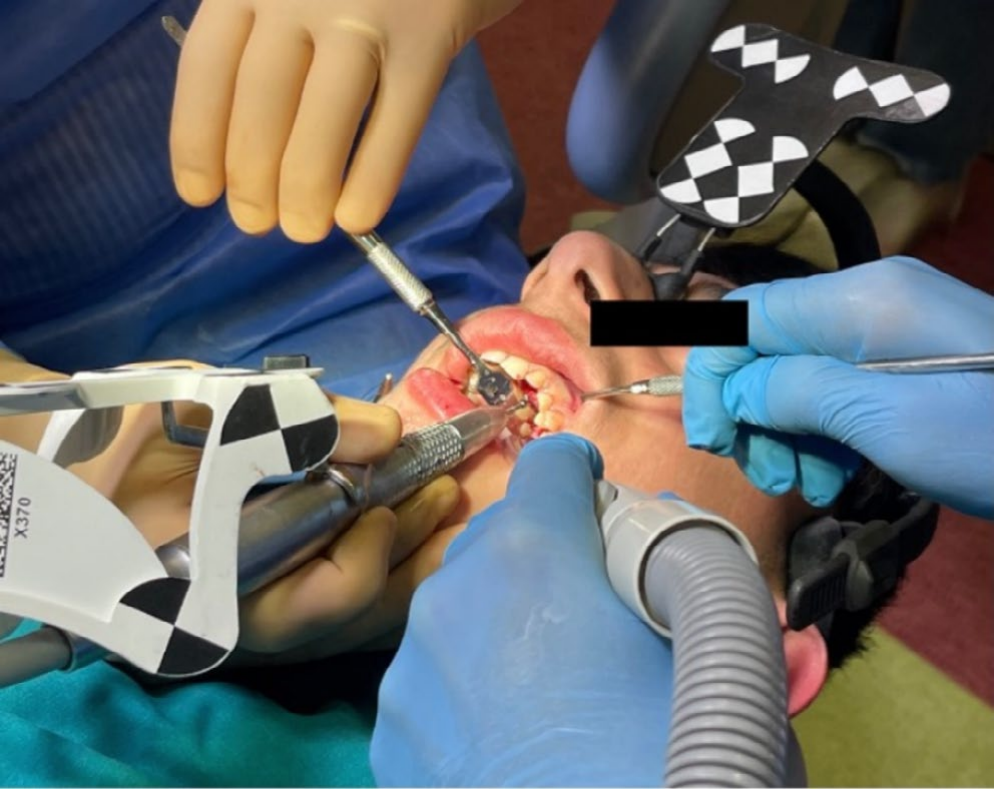
Execution of guided osteoctomy.

**FIGURE 10:**
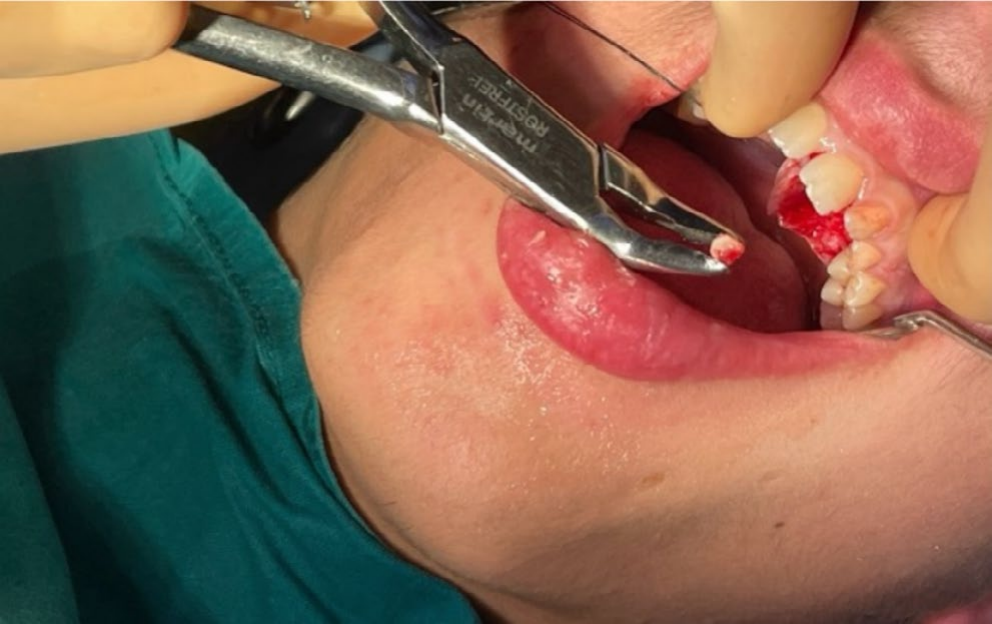
Photograph during the removal of the odontoma.

**FIGURE 11:**
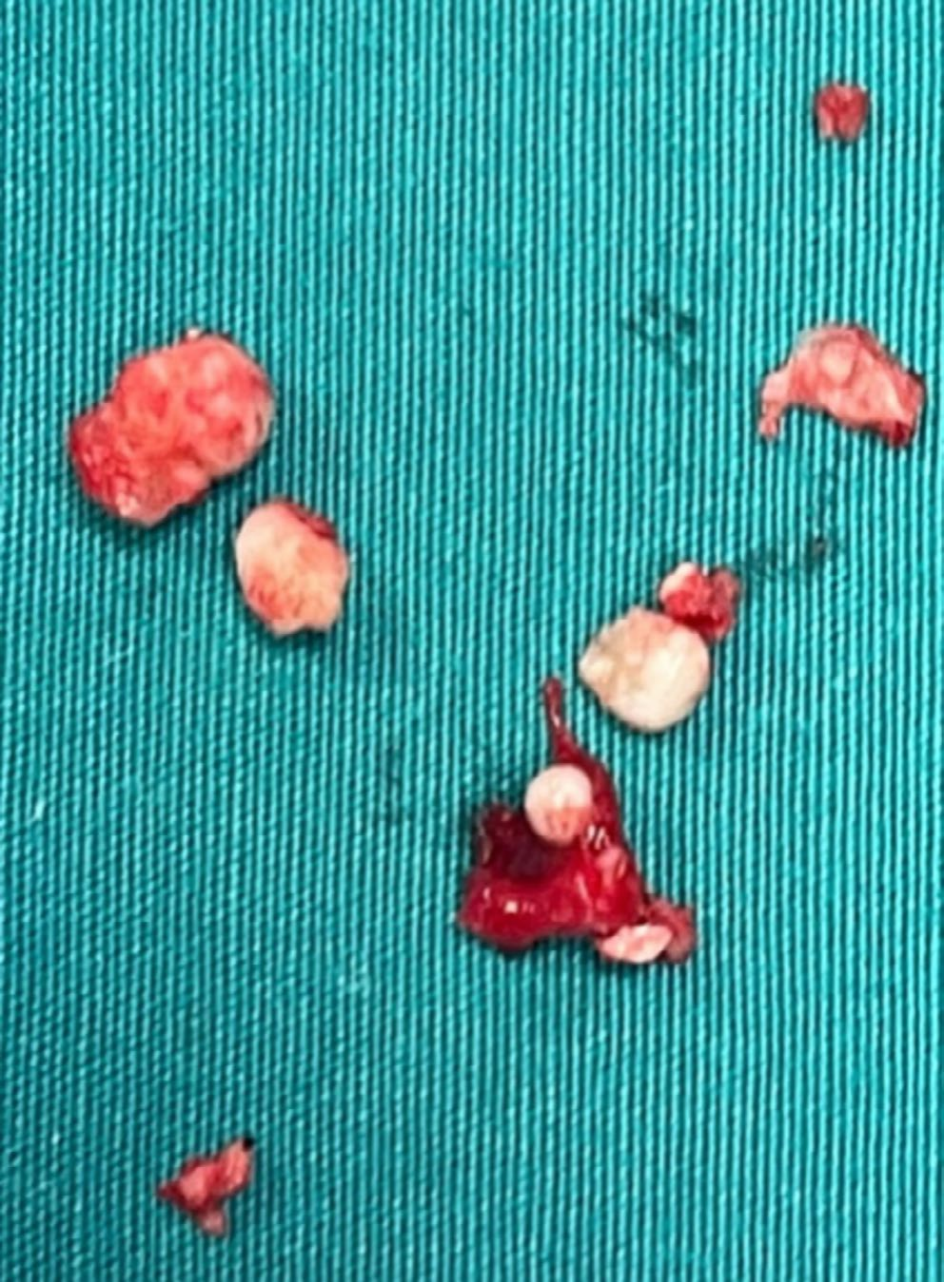
Photograph after the composed odontoma removal.

An ostectomy was then performed to first reveal the composed odontoma (Figure 9) and subsequently, the impacted tooth. The removal of the odontoma was guided by the Navident system (Figures 10 and 11).

**FIGURE 12:**
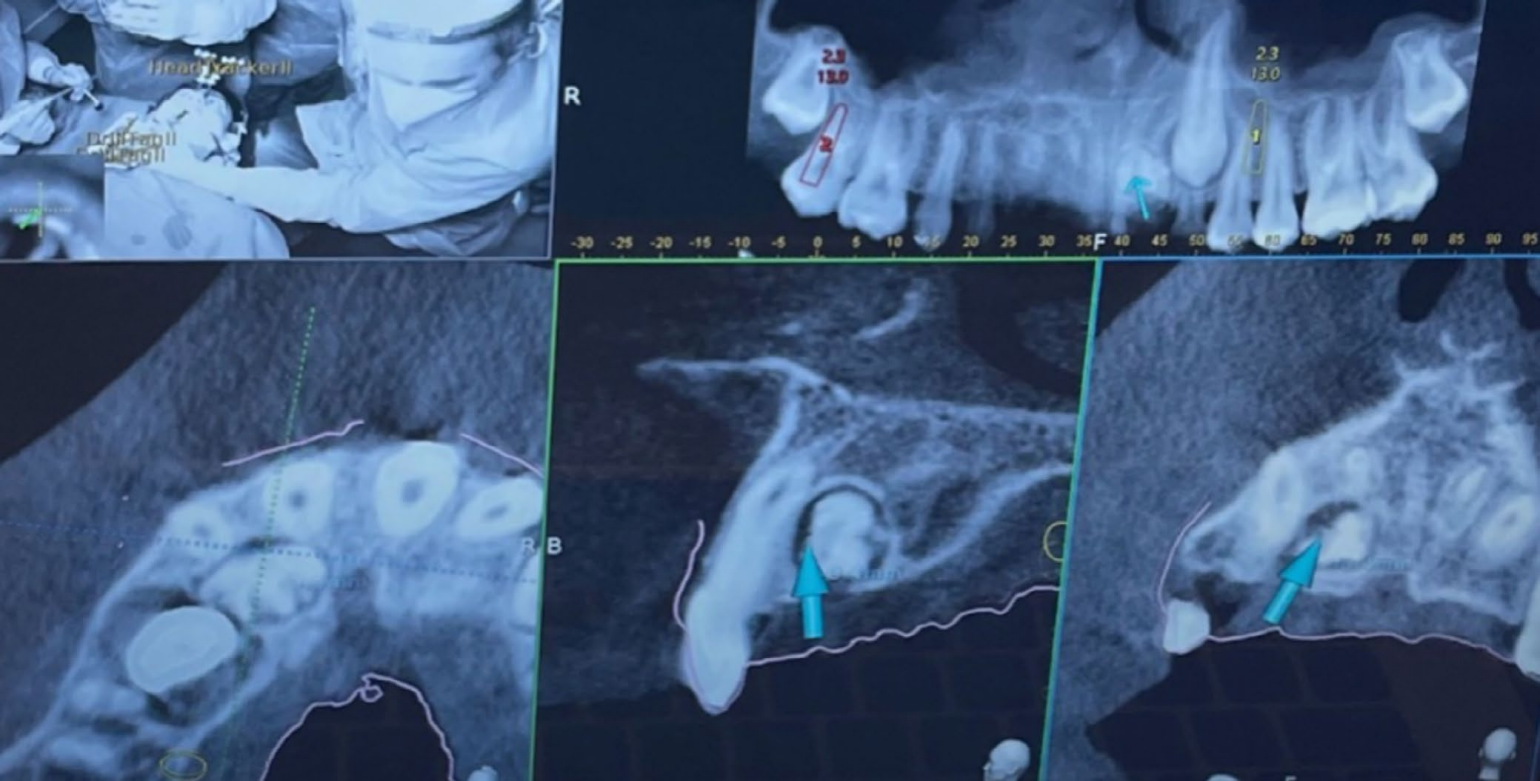
Navigation view during the surgical cavity inspection using the Navigation system.

**FIGURE 13:**
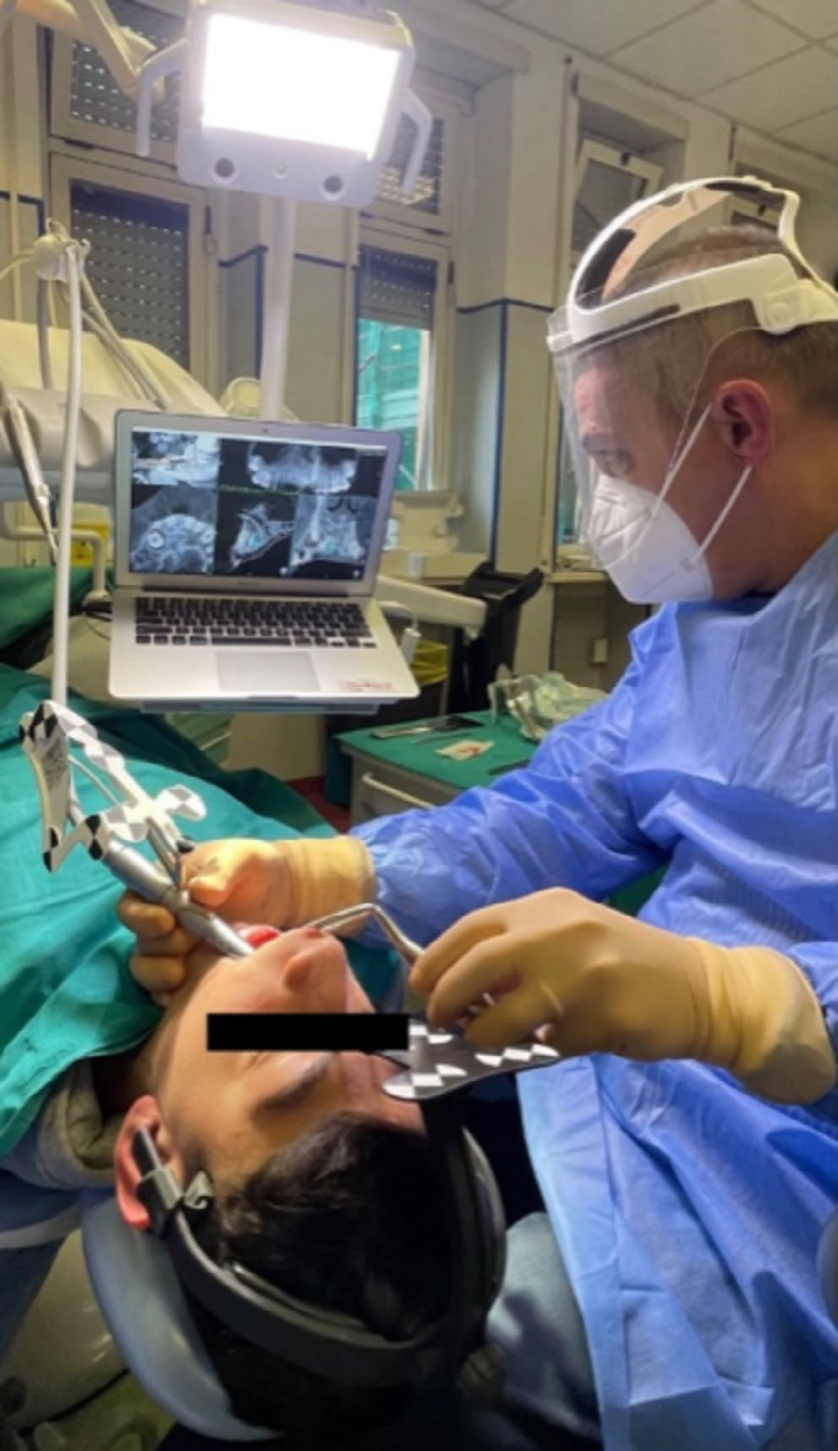
Surgical cavity inspection using the Navigation system.

The deciduous canine was left in place to preserve interdental space and maintain bone thickness. After enucleation, the surgical cavity was carefully inspected using the dynamic tracking capabilities of the Navident system, which allowed real‐time monitoring of the instrument's tip position in space, ensuring precise assessment of the cavity boundaries (Figures 12 and 13).

**FIGURE 14:**
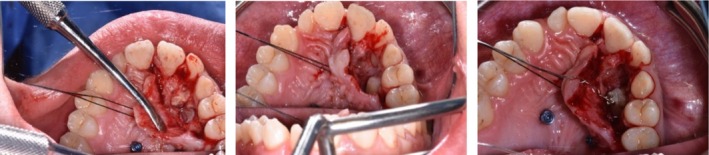
Occlusal view showing the inserted miniscrews and the flap to expose the surgical area of interest after odontoma removal.

**FIGURE 15:**
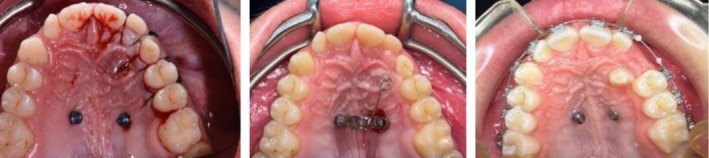
Evolution of the guided eruption of the upper canine.

**FIGURE 16:**
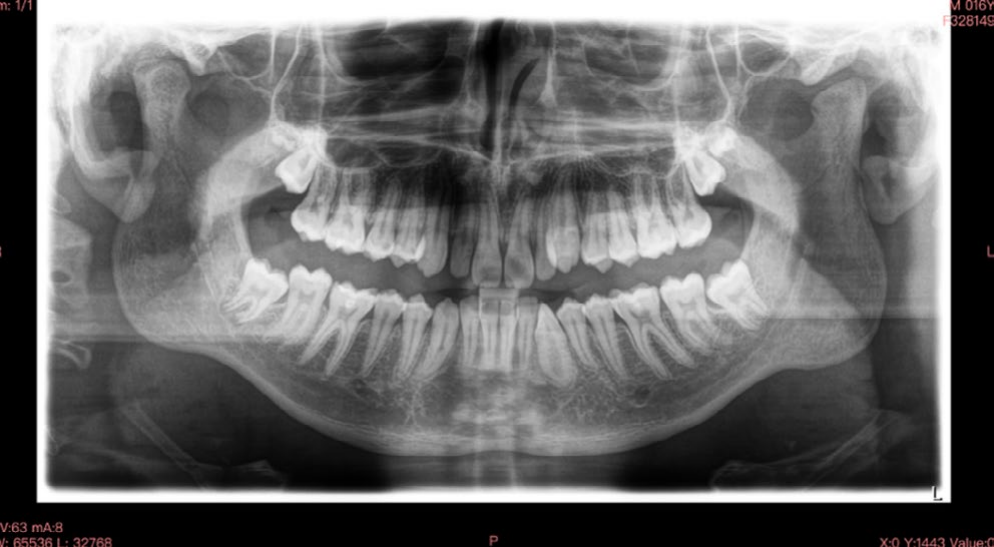
Orthopantomography at 13 months follow‐up showing the eruption of the canine and absence of odontoma relapse.

**FIGURE 17:**
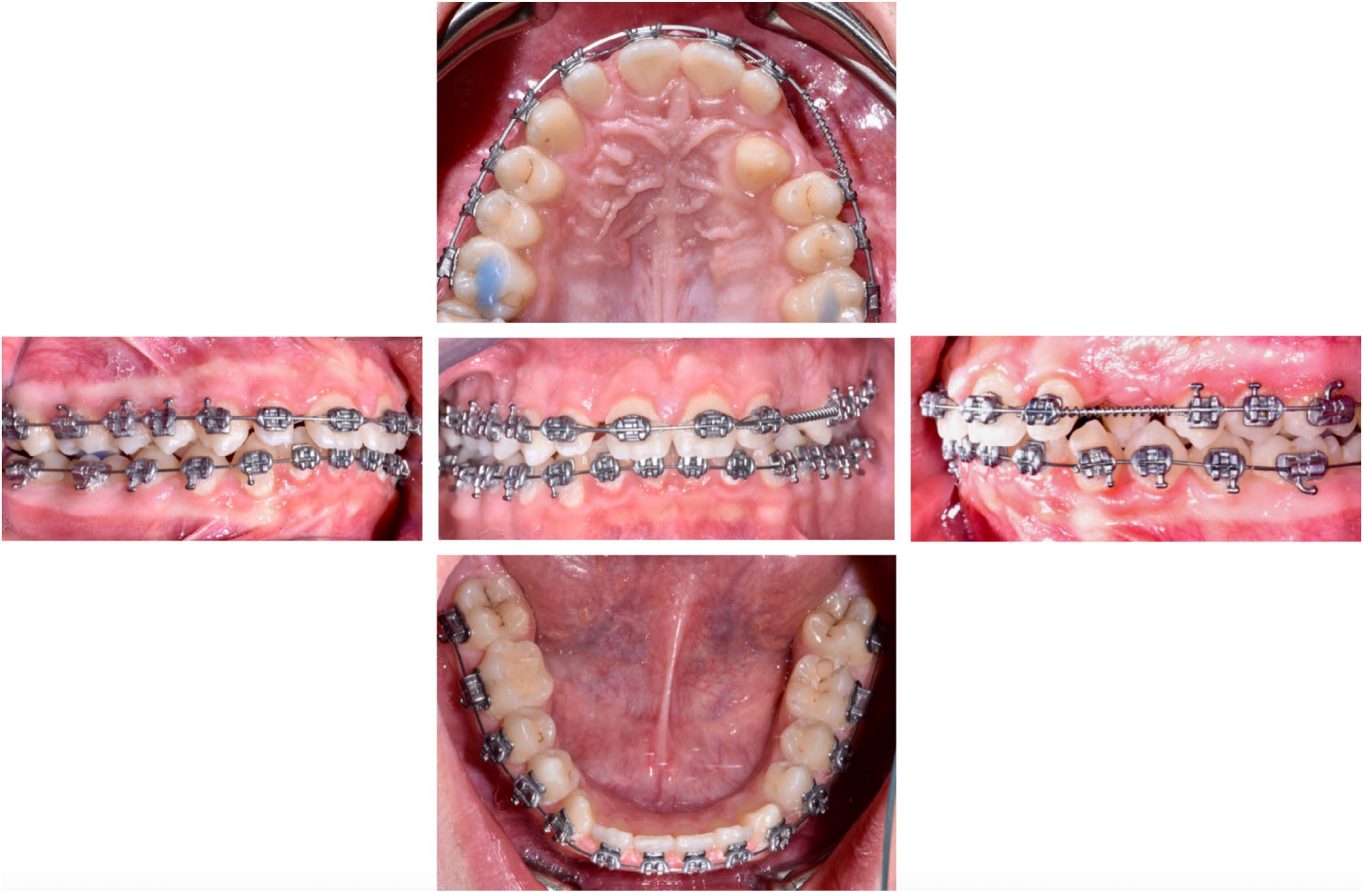
Intraoral photographs at 13 months follow‐up since the surgical intervention and start of orthodontic treatment.

The surgical phase concluded with suturing the surgical flap using Vicryl 3.0 thread (Vicryl Ethicon, Johnson & Johnson, Somerville, NJ, USA) and with the activation of palatal canine traction, which was continued in the following weeks until eruption (Figures 14, 15, 16, 17).

### 5.3 Treatment Results

After the surgical phase, the patient did not show severe difficulties, the wound healed without complications, and the mini‐screw insertion was performed without complications. The orthodontic treatment successfully repositioned the canine into the arch, and finally, an orthodontic finalization through a fixed multibracket appliance was necessary to restore the correct occlusal relationships.

At the end of treatment, the radiographic examination showed no recurrence of the odontoma, demonstrating an accurate removal of the lesion.

## 6 Discussion

Odontomas, although generally benign, present significant clinical challenges due to their impact on dental development and tooth eruption. Their asymptomatic nature often results in delayed diagnosis, which typically occurs only when routine imaging is performed, or when clinical signs, such as delayed tooth eruption or swelling, prompt further investigation [1, 2].

Compound odontomas are more commonly found in the anterior maxilla, whereas complex odontomas frequently appear in the posterior mandible [1, 2, 3]. Studies reveal that early intervention is critical, as the presence of odontomas can disrupt the development of adjacent teeth as documented in the case here described [4, 5, 6].

Surgical excision remains the primary treatment modality and is generally successful in eliminating barriers to eruption while preventing potential complications [3, 4, 5]. However, in some cases, additional orthodontic intervention may be required, particularly when odontomas cause displacement or rotation of neighboring teeth [3, 6, 7, 8]. Due to the rare but possible recurrence of odontomas, periodic follow‐up is recommended to ensure complete resolution and to monitor any changes in dental alignment [6, 7, 8, 9, 10].

The main challenge in the case presented here stemmed from the anatomical proximity between the odontoma and the permanent teeth, which put the latter at significant risk of radicular‐apical damage during the surgical procedure to remove the odontoma, particularly since it was also decided to attempt the recovery of the impacted canine.

For this reason, the decision to use a dynamic navigation system was a crucial operational choice. It allowed us to enhance the accuracy of the intervention, not only relying on CBCT data but actively interfacing with the system in real‐time during the surgery [12].

Furthermore, the use of this technology facilitated an easy post‐surgical revision of the extraction site, eliminating the need for post‐operative x‐rays, instead of utilizing the handpiece insert as an inspection probe. The necessity of a delicate and highly precise approach to odontomas is well established in the scientific community, with other researchers advocating for the use of CAD‐CAM technology to manage such clinical situations [10].

Although dynamic navigation systems were initially introduced in clinical practice primarily for implant‐prosthetic rehabilitation [11], particularly in complex cases [13], their evolution, while still regarded as a niche technology, has greatly expanded their range of applications. Today, official protocols exist that are specifically tailored for non‐implant procedures [17, 21].

This progress has been facilitated by both clinical experience and the development of supports and adapters for non‐implant handpieces. In this case, the combination of dynamic navigation and non‐implant handpiece instruments provided a clear advantage.

In recent years, the evolution of dentistry has extended beyond digitalization, with an increasing shift towards robotics and artificial intelligence. As a result, the term “robotic dentistry” or “dentistry based on artificial intelligence technologies” has become more relevant than “digital dentistry” [12, 22].

The accuracy of implant positioning and surgical procedures largely depends on the experience and manual skill of the clinician. Even minor deviations during the placement procedure can lead to functional or aesthetic complications such as nerve damage or implant failure. According to some authors, deviations can result in implant failure rates of up to 10% even among the most experienced surgeons [10].

While computer‐assisted static and dynamic implant surgery improves the accuracy of implant or orthodontic miniscrew placement compared to freehand procedures [22, 23], these methods still have inherent limitations. In static computer‐assisted surgery, for example, the guide must be produced before surgery and cannot be adjusted intraoperatively [24, 25].

Dynamic systems offer greater flexibility by providing real‐time feedback and the ability to modify the surgical plan during the procedure. These features present significant advantages over static systems. However, dynamic systems have some limitations since they still require the surgeon's continuous attention to the screen, rather than direct observation, which can introduce errors due to fatigue or manual inaccuracies; further, these systems are characterized by high initial costs, a learning curve, and finally are subject to the availability of equipment [16, 26, 27]. Consequently, the latest advancements in robotic and artificial intelligence‐assisted dentistry are focused on automating the entire surgical phase through Autonomous Dental Implant Robotic Systems (ADIRS), which are pre‐programmed and do not require integration with the clinician's hand [28].

In our experience, however, to date, the use of fully automated surgical systems remains risky, and the management of complex cases should be based on the integration of experience, clinical expertise, and technological innovation. While automation continues to evolve, the surgeon's role remains essential in decision‐making and adapting to patient‐specific anatomical variations. In this area, dynamic navigation still has substantial untapped potential. The progressive refinement could further improve precision, making it an indispensable tool for both routine and complex oral surgery procedures.

## 7 Conclusions

This case‐report highlights the effectiveness of dynamic computer‐guided surgery in the removal of an odontoma near critical structures, such as the permanent incisors and impacted canine. The use of dynamic navigation with CBCT allowed for precise planning and real‐time guidance, minimizing risks and ensuring a safer procedure. This technology not only enhanced accuracy but also eliminated the need for post‐operative radiographs. Dynamic navigation systems proved crucial in managing complex cases like this, offering significant potential for improving the precision and safety of oral surgeries in the future.

## Author Contributions

**Martina Mezio:** data curation, writing – original draft, writing – review and editing. **Davide Brilli:** data curation, writing – original draft, writing – review and editing. **Alessandra Putrino:** data curation, writing – original draft, writing – review and editing. **Matteo Giansanti:** software, writing – review and editing. **Michele Cassetta:** conceptualization, supervision, writing – review and editing.

## Consent

Written informed consent has been obtained from the patient's parents.

## Conflicts of Interest

The authors declare no conflicts of interest.

## Data Availability

The data that support the findings of this study are available from the corresponding author upon reasonable request.
